# Cystic Anomaly Manifestation Within the Axillary Fossa: A Case Report of Clinical Significance

**DOI:** 10.7759/cureus.61200

**Published:** 2024-05-27

**Authors:** Vladimir Aleksiev, Dimcho Argirov, Boyko Yavorov, Daniel Markov, Filip Shterev

**Affiliations:** 1 Department of Cardiovascular Surgery, Medical University of Plovdiv, Plovdiv, BGR; 2 Department of Surgery, Medical University of Plovdiv, Plovdiv, BGR; 3 Department of General and Clinical Pathology, Medical University of Plovdiv, Plovdiv, BGR; 4 Department of Internal Diseases, Section of Pneumonology and Physiatrics, Medical University of Plovdiv, Plovdiv, BGR

**Keywords:** bible cyst, paralabral cyst, glenohumeral cyst, ganglion cyst, axillary mass

## Abstract

Ganglion cysts represent a small group of lesions that can arise from almost any joint in the body. Demonstrating a predilection for the joints in the hand and wrist, ganglion cysts in the glenohumeral joint are extremely rare. Due to the vivid array of masses that can be found in the axillary fossa, forming a free-from-error work-up to the correct diagnosis can be quite confounding. In this paper, we present a case of a paralabral cyst of the shoulder joint, located in the axilla. With there being only eight other such cases published in clinical literature, we believe this case report to be of unique importance in gaining further insight into the genesis and treatment of this pathology.

## Introduction

A paralabral cyst or ganglion cyst, colloquially known as a Bible cyst, is a benign bulge that appears around joints and tendons. In its essence, a ganglion is a sack filled with synovial fluid, protruding from a joint’s capsule of muscle. This tear further dissects and extends into the surrounding connective tissue as it enlarges and grows. The main culprit in the pathogenesis of their formation is acute trauma or repetitive motion (chronic microtrauma). The most common site of a ganglion cyst appearing in the margins of the glenohumeral joint is the posterosuperior aspect of the joint which is accounted for by the fact that the posterosuperior capsule above the posterior band of the inferior glenohumeral ligament is an area of relative weakness when compared to the thicker anterior capsule [1,2]. This pathology is three times more common in men in their third and fourth decade and is accounted for in about 2-4% of the general population. Cysts arising from the inferior portion of the joint are extremely rare.

Intrinsically a ganglion cyst is not a cyst per se, abiding by its definition, which states that a true cyst’s cavity Is lined with epithelium. A synovial cyst is lined with synovial cells and forms from the evagination of the joint capsule or para-articular bursa. A pseudocyst is lined with fibrous tissue and is filled with mucoid content. A true ganglion cyst may arise from the joint capsule, bursa, ligament, tendon, or subchondral bone and is a result of myxoid degeneration of the connective tissue of the joint capsule.

Their size can vary over time and some cysts even disappear completely on their own as a result of the labial tear healing over time. Clinically a patient presents with localized pain, especially in the early stages of cyst formation. The level of pain does not correlate with the size of the cyst.

Most ganglion cysts are found incidentally during routine imaging. They rarely become clinically evident unless they cause compression of surrounding structures and mass effect. A review of orthopedic literature reveals that ganglion cysts of the shoulder mostly present with symptoms of entrapment or compression of the suprascapular or axillary nerve [3]. Larger cysts in the shoulder area, which present with little to no symptoms are exceedingly rare.

## Case presentation

**Figure 1:**
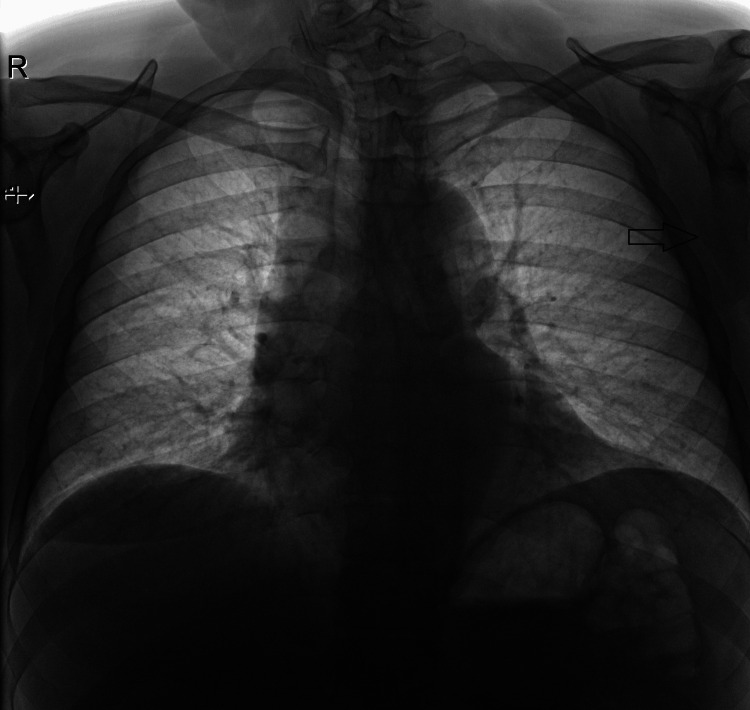
Pre-operative X-ray revealing a shadow in the left axilla

**Figure 2:**
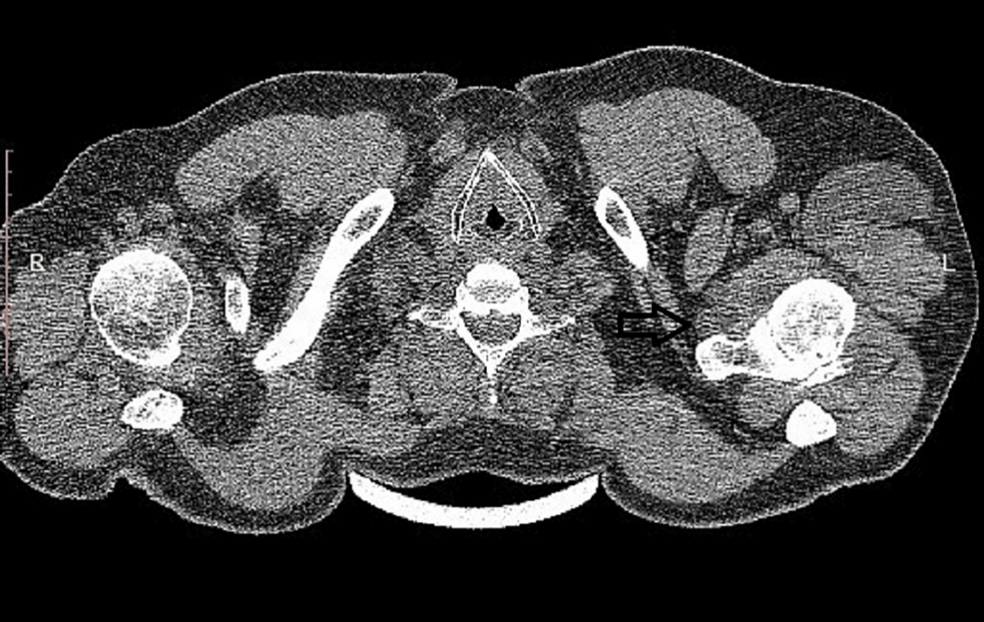
CT image showing the glenohumeral joint, giving rise to the cyst

**Figure 3:**
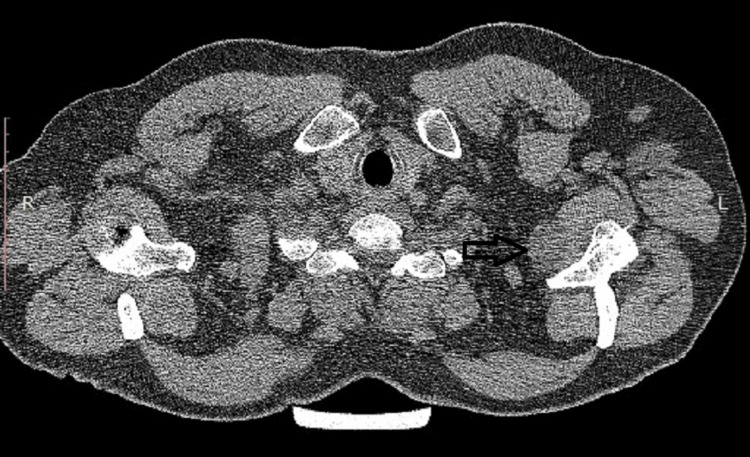
CT image of the cyst as it descends further into the soft tissues

**Figure 4:**
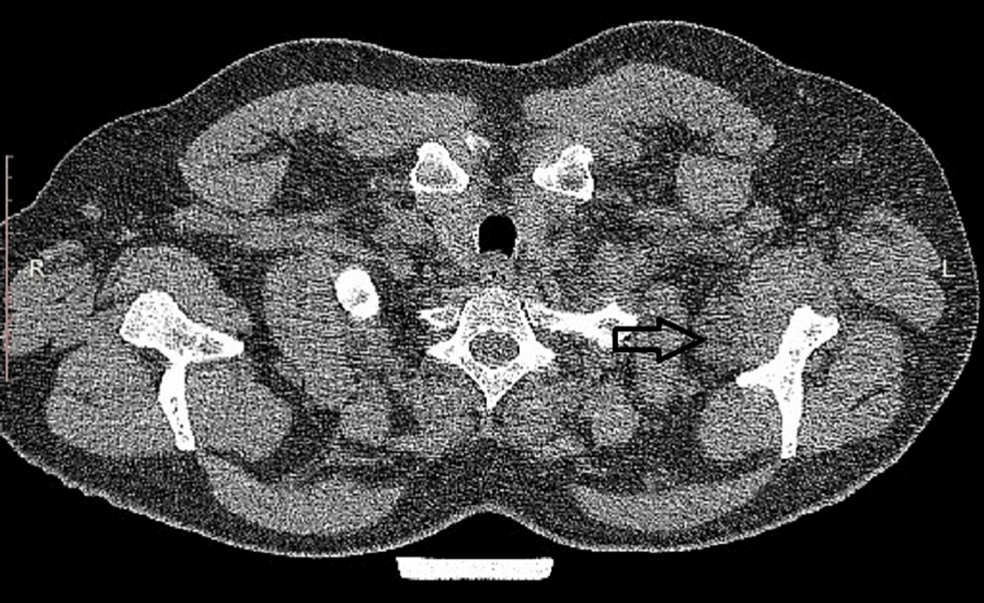
Final image from pre-operative CT showing the ganglion cyst as it extends almost 5 cm downwards into the axillary fossa

A 52-year-old man was admitted to the thoracic surgery clinic with complaints of a mass in the right axilla which had grown over the span of a few weeks. He reported no relevant medical history and remained asymptomatic up to the time of admission. Physical examination revealed a palpable mass in the right axillary fossa. It measured around 5cm in diameter and was rubbery to the touch, non-tender, and was not fixed to the surrounding tissues. The CT scan showed a 7 cm cylindrical mass with sharp edges, which was in intimate relation to the glenohumeral joint and was well-defined from adjacent tissue (Figures 1-4).

**Figure 5:**
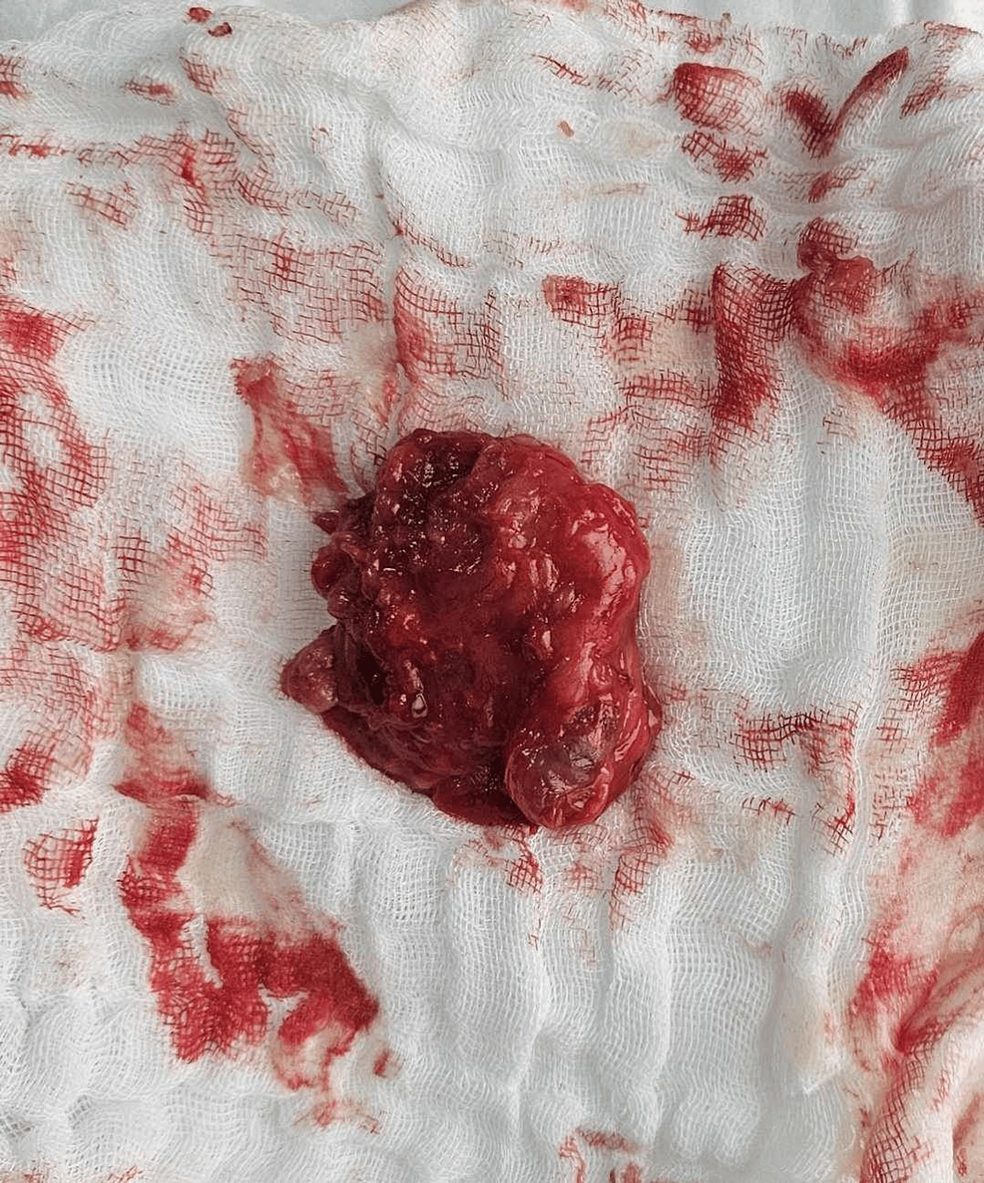
Ganglion cyst after surgery

An axillary lipoma was suspected and based on the clinical examination, imaging, and the patient’s recent history a decision was made to remove the mass. Upon careful dissection, it was evident that the mass was adjacent to the inferior border of the glenohumeral joint. This led the team to believe that this was in fact a large ganglion cyst. The cyst was removed, and the labrum was inspected. No evidence of tear or wear was noted. The mass was filled with gelatinous fluid and post-operative histology confirmed the intraoperative diagnosis (Figure 5). A drain was placed and was removed after 48 hours. The patient experienced an uneventful postoperative period and was discharged on the second day after surgery. He reported no issues upon follow-up.

## Discussion

In an attempt to illustrate the wide differential diagnosis of axillary masses, a study was carried out by de Andrade et al., evaluating 31 patients with isolated axillary masses [4]. Nine patients were diagnosed with occult breast cancer, five of them in the contralateral breast. Seven had metastatic lymph nodes of non-ductal origins, one had carcinoma of apocrine cells with metastasis to the axilla. Four patients had benign lymphadenopathy and four had ruptured infundibular follicular cyst, nodular fibromatosis inflammatory tuberculous, and inflammatory rheumatoid lymphadenitis. Five had an ectopic breast and one patient presented with an axillary lipoma.

Following this line of thought, when treating tumors in the axillary fossa one should exclude soft tissue sarcomas, leukemia, lymphoma, blocked and/or inflamed hair follicles, boils and carbuncles, epidermal inclusion cysts, ingrown hairs and folliculitis, mononucleosis any all causes of lymphadenitis.

The diagnosis of ganglion cysts can be made fully relying on the patient’s history and physical examination. If any kind of wear or trauma is suspected or if there is the slightest probability of malignancy, additional investigation is warranted. Ultrasound and X-ray are good tools, with ultrasound being the easiest and most common way to visualize this pathology. MRI is stated to have a sensitivity of up to 80% and a specificity of about 50% when it comes to ganglion cysts [5].

Most ganglion cysts can disappear without treatment (38-58%) [6,7]. Invasive procedures include aspiration with the intent of draining the cyst’s content and decompression, with or without the introduction of sclerosing agents. Decompression can alleviate symptoms but repeat aspiration is cited to have a success rate of only 30-50% [8,9]. On the other hand, surgery shows better curative results as compared to aspiration/injection. Excision remains the gold standard for treatment. It is somewhat debatable whether simple excision is sufficient or if any further stabilization or repair should be carried out. Our experience shows that if there is no evident labral tear or instability, excision of the cyst is more than sufficient.

## Conclusions

In conclusion, paralabral cysts in the inferior aspect of the glenohumeral joint can present a diagnostic difficulty when it comes to treating masses in the axillary fossa. Diagnosis can be achieved with a thorough physical examination, rigorous history-taking, and an adept ultrasound technician. Excision of the cyst with labral repair when needed still remains the treatment of choice when treating this pathology.
